# Effect of Carbamazepine, Ibuprofen, Triclosan and Sulfamethoxazole on Anaerobic Bioreactor Performance: Combining Cell Damage, Ecotoxicity and Chemical Information

**DOI:** 10.3390/toxics10010042

**Published:** 2022-01-17

**Authors:** Mabel Díaz-Cubilla, Pedro Letón, Carlos Luna-Vázquez, Marta Marrón-Romera, Karina Boltes

**Affiliations:** 1 Departamento de Química Analítica, Química Física e Ingeniería Química, Universidad de Alcalá, Ctra. Madrid-Barcelona Km. 33,600, 28871 Alcala de Henares, Spain; mabel.diaz@edu.uah.es (M.D.-C.); pedro.leton@uah.es (P.L.); 2 IMDEA Water Institute, Parque Científico Tecnológico, 28805 Alcala de Henares, Spain; 3 Departamento de Electrónica, Universidad de Alcalá, Ctra. Madrid-Barcelona Km. 33,600, 28871 Alcala de Henares, Spain; carlos.luna@uah.es (C.L.-V.); marta.marron@uah.es (M.M.-R.)

**Keywords:** anaerobic, pharmaceuticals and personal care products, toxicity, cell damage, enzymatic activity, image analysis

## Abstract

Pharmaceuticals and personal care products (PPCPs) are partially degraded in wastewater treatment plants (WWTPs), thereby leading to the formation of more toxic metabolites. Bacterial populations in bioreactors operated in WWTPs are sensitive to different toxics such as heavy metals and aromatic compounds, but there is still little information on the effect that pharmaceuticals exert on their metabolism, especially under anaerobic conditions. This work evaluated the effect of selected pharmaceuticals that remain in solution and attached to biosolids on the metabolism of anaerobic biomass. Batch reactors operated in parallel under the pressure of four individual and mixed PPCPs (carbamazepine, ibuprofen, triclosan and sulfametoxazole) allowed us to obtain relevant information on anaerobic digestion performance, toxicological effects and alterations to key enzymes involved in the biodegradation process. Cell viability was quantitatively evaluated using an automatic analysis of confocal microscopy images, and showed that triclosan and mixed pollutants caused higher toxicity and cell death than the other individual compounds. Both individual pollutants and their mixture had a considerable impact on the anaerobic digestion process, favoring carbon dioxide production, lowering organic matter removal and methane production, which also produced microbial stress and irreversible cell damage.

## 1. Introduction

Anaerobic digestion (AD) is a formal treatment technology used to treat high organic load wastewaters and the sewage sludge generated by Wastewater Treatment Plants (WWTP). Although at its inception, the aim of this treatment was organic matter removal, nowadays it is used for maximizing methane production, becoming a priority goal in its design and operation. Both methane and digested sludge are valuable resources under the novel concept of a circular economy. In this new economic model, the maintenance of the value of materials and energy is desired during the different productive cycles, in order to minimize waste production and resource consumption [1].

Interest in AD relies on its potential ability to transform the energy contained in wastewater (organic matter) into methane, for its later use as an energy vector for heat generation, automotive fuel, and injection into the natural gas network. There are several recent works focused on this process optimization to enhance methane production by using different strategies, i.e., pre-treatments [2,3], thermal hydrolysis highlighting [4], microaerobic conditions management, [5] use of enzymes [6] and microwaves [7], or use of additives [8] such as biochar [9]. However, other works focused on hydrogen production [10].

On the other hand, the so-called Contaminants of Emerging Concern (CEC) which describes many daily used compounds, have recently been detected both in industrial and domestic wastewater as well as in different environmental compartments. Pharmaceuticals and Personal Care Products (PPCPs) is one of its main families due to its number, concentrations, and negative effects on ecosystems [11]. The concentration of these pollutants in wastewater is usually very low (micro or nanograms per liter), but their continuous accumulation may cause toxicological and other environmental impacts, as they have just been only noticed during the last decades. 

Conventional WWTP are not designed for the optimal removal of CEC. Recently, studies in the literature analyzed PPCPs in WWTP, based on mass balances and considered their biological or abiotic degradation, presence in liquid phase effluents, or sorption in the sludge sewage [12,13], even focusing on AD of sewage sludge [14]. The analysis of sludge samples from several WWTPs showed that nearly 20 PPCPs can be detected in sewage sludge in concentrations up to 100 ng/g. [15,16]. Frequently, mass balance is not clearly obtained because of the limitations of analytical techniques, especially for the determination of contaminants retained in its solid phase. [17,18].

Many works also reported anaerobic biodegradation of PPCPs, obtaining dissimilar results regarding their removal efficiency [19,20,21]. There are many studies related to the toxicity and potential risk associate with the presence of these substances in natural environments [22,23], but more limited is the information published on how the presence of these substances affects the biological treatment process itself. 

For aerobic processes, the toxicological damage caused by PPCP to typical enzymatic activities was studies, in order to determine the response of the microbial population against toxics both with single compounds [24,25] and mixtures [26]. In all cases, PPCPs were found to cause an alteration in the process performance to different degrees.

Considering the capacity of biological sludge to retain PPCPs, it becomes a priority to investigate the way these pollutants affect the overall anaerobic process, as AD is the main process used for sludge stabilization in WWTP around the world. Recently, Azizan et al. [27] reported that most of the PPCPs induce the accumulation of Volatile Fatty Acids (VFA), causing a decrease in both the methane production and substrate utilization. PPCPs adsorbed in sludge also modify the microbial ecology of bioreactors, increasing the population of Firmicutes and decreasing that of Bacteroidetes and Euryarchaeota. This ecological changes in anaerobic reactors can also cause VFA accumulation [28]. 

Although anaerobic bacterial populations are sensitive to different toxics, there is little information on the effect that pharmaceuticals exert on their metabolism. Moreover, microbial management of bioprocesses is an emerging topic with great potential, particularly for AD involving a huge biodiversity [29].

In our previous works [26,30], we reported the toxic effects of the anti-inflammatory drug ibuprofen (IBU) and the antimicrobial agent triclosan (TCS) on the microbial activity of aerobic activated sludge. In these previous articles, the negative effect of pharmaceuticals was evaluated via an assessment of cell viability, respiration rates and changes to key enzymatic activities, as a response to oxidative damage caused by selected contaminants. However, the aim of those study is to evaluate the individual and combined effect of four PPCPs on the activity of microorganisms in anaerobic digester, attending for first time to their influence on some key enzymes involved in organic matter uptake and detoxification processes, as well as to the cell viability in the microbial consortium. 

For this study, we selected IBU, TCS, the antibiotic sulfamethoxazole (SFM) and the anticonvulsant carbamazepine (CBZ). They belong to most representative’s groups of PPCPs because of their widespread use. The occurrence of these organic micropollutants in water bodies, influents, and effluents of WWTPs on a global scale, is well-documented. Most recent published works [31,32,33,34] revised the information available on their negative effects, and how they impacted on aquatic organisms, showing toxicity on different taxa as well as bioaccumulation. With regard to the environmental risk, all of them are candidates for inclusion in the “watch list” of substances for European Union-wide monitoring in the field of water policy, with SFM finally included in the top ten substances [35].

The focus at the enzymatic level is on the production of Oxygen Reactive Species (ROS), which cause oxidative stress in biological reactors, as well as on the enzymes associated with the control of oxidative stress and the detoxification process, namely catalase (CAT), glutathione s-transferase (GST) and the esterase (EST). 

EST is a hydrolytic enzyme that catalyze the first stage of anaerobic digestion through catabolic (destructive) processes [36]. GST is involved in Phase II of the detoxification processes of several organics, thereby increasing the hydrophilic nature of xenobiotic to facilitate their elimination from the cell [37]. ROS include different compounds, ionic and radical species with greater reactivity and redox activity than molecular oxygen [38]. When stress situations occur, for example in presence of toxic substances, an overproduction of ROS takes place tending to eliminate these chemicals from cell. This can cause an imbalance due to the accumulation of ROS, which finally promote oxidative stress process, a very damaging situation for cells because it leads to the oxidation of biomolecules, irreversible cell damage and cell death [37]. The enzyme CAT is primarily responsible for ROS cleavage, and an increase in CAT activity is associated with cell stress due to the accumulation of ROS in the presence of a toxic substance. These enzymatic responses, and the oxidative stress caused by pollutants are well described in several aquatic and terrestrial organisms such as fish [39], plants [40] and invertebrates [41,42]. Nevertheless, changes in enzymatic activities and ROS generation, in response to the presence of pharmaceutical compounds, have not been evaluated so far in anaerobic bacterial communities. In fact, in AD technology, researchers are focused on identifying specific bacteria and enzymes involved in the transformations of selected organic micropollutants. Recently, Gonzalez-Gil et al. in [43] reviewed available information on the mechanism for anaerobic degradation of several organic pollutants, but authors conclude that it is very difficult to predict the biotransformation pathway also identify all the enzymes involved, due to the very complex molecular structure of pollutants.

In the same way, another objective of this work was to determine if the selected pollutants caused irreversible cell damage and how the cell viability relates to alterations of the enzymatic activities measured. For the analysis of cell viability, we used for first time a quantitative methodology which permits the evaluation of viable and nonviable cells from images obtained by confocal microscopy. 

This work aims to provide further insights into the toxicity mechanisms induced by individuals and mixed PPCPs pollutants to the microbial activity of biomass in anaerobic digestion.

## 2. Materials and Methods

### 2.1. Wastewater and Micropollutants

Synthetic wastewater (SW) containing nutrients was prepared in distilled water, based on the recipe provided in [44]. All chemicals were of analytical grade. This wastewater was used during the operation of aerobic and anaerobic bioreactors for biomass production.

The following PPCPs were added to the SW for the exposure test: sulfamethoxazole (CAS 723-46-6), carbamazepine (CAS 298-46-4), ibuprofen (CAS 15687-27-1), triclosan (CAS 3380-34-5). All these chemicals were of 99% purity and supplied by Sigma-Aldrich.

Micropollutants were dissolved in methanol and maintained in concentrated stock solution of 2000 mg/L and −20 °C until use.

The following reagents were purchased from Sigma-Aldrich and were used to determine enzymatic activities: monochlorobimane (MCB), 2′,7′-dichlorofluorescin diacetate (H2DCF-DA), reduced glutathione (GSH) and the solvent dimethyl sulfoxide (DMSO, 99.9%).

Cell damage was evaluated using Live/Dead Backlight Bacterial Viability staining purchased from Molecular Probes, Invitrogen.

### 2.2. Bioreactors and Experimental Procedure

The first step was the production of biomass under controlled conditions. For this, aerobic sludge was generated using a 5 L aerated bubble column laboratory reactor coupled with a sedimentation vessel for withdrawn biomass. The aerobic system was fed with SW at 0.5 days HRT, following the same procedure reported by Amariei et al., 2017 [30].

In the second step, for the anaerobic inoculum production, the excess of aerobic sludge was recovered every three days. The collected sludge was incubated during 120 days under anaerobic conditions in a 2.5 L agitated reactor (37 ± 3 °C, pH = 7.0 ± 0.5) operated in a semicontinuous mode. SW and sludge were added at intervals of three days in order to maintain TOC = 1700 ± 500 mg/L, according to OECD Test Guide 209 [45]. Biogas generation was measured daily, determining also organic matter as well as TOC by TOC-VCSH Shimadzu, and the methane and carbon dioxide (CO_2_) content by GC-TCD Varian 3350, according to [46]. When the anaerobic reactor for inoculum production reached stable operation (monitored by biogas production and TOC removal), anaerobic biomass was ready to be exposed to the selected micropollutants.

**Scheme 1:**
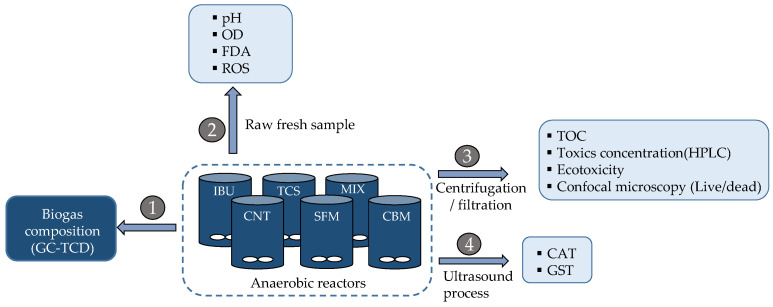
Experimental setup and sample treatment for chemical and biochemical analysis.

In the third step, the exposure experiments of anaerobic biomass to PPCPs were carried out in batch conditions. Here, six agitated reactors of 400 mL reaction volume were operated in parallel. The experimental setup included one control (reactor without PPCPs); four reactors containing 1 mg/L of single PPCPs and one reactor containing a mixture of 1 mg/L of selected PPCPs (see Scheme 1 for details). All reactors permitted biogas monitoring and liquid sampling. 

Over three days, bioreactors were sampled for (a) chemical analysis (concentration of toxics by SPE + HPLC-DAD and organic matter as TOC); (b) measurement of enzymatic activities (esterase, GST, CAT, ROS generation); (c) cell damage (double staining of cells, confocal microscopy, and image processing); and (d) ecotoxicity on green algae. 

### 2.3. Analytical Techniques, Ecotoxicity and Enzymatic Activity

The chemical analysis included measurement of the toxics (each PPCPs) in liquid and solid fractions of anaerobic reactors in the assays. Samples were processed according to the protocol detailed in Scheme 1. There, it is shown the different parameters obtained from the fractions of liquid samples (arrows 2, 3 and 4), as well as their previous treatment. Gas phase (arrow 1) indicates the chromatographic analysis of biogas composition. 

PPCPs were determined by HPLC-DAD, according to the method developed in this work. For this, an Agilent LC 1260 system under isocratic elution was used and operated at ambient temperature. The eluent was a 50:50 volume proportion acetonitrile: water (with 0.1% H_3_PO_4_) at 0.5 mL/min. Separation was done by ODS Hypersil C18 column (4.6 mm × 150 mm, 5 μm). Detection was carried out at 280 ± 4 nm (SFM and CBM) and 210 nm ± 4 nm (IBU and TCS). Under these conditions, a calibration of the proposed method was carried out using 7 concentrations of external standards. 

The TOC concentration in liquid phase and biogas production and composition were monitored according to the same methods described in Section 2.2.

Ecotoxicity was evaluated using the green algae *Raphidocelis subcapitata*, following Test Guide 201 [47] with the modifications reported in [48].

ROS, and GST activities were determined according to the procedure described by Amariei et al., 2020 in [26]. Briefly, GST activity was evaluated by the reaction of MCB, while ROS was measured by the fluorescence H2DCF-DA. Esterase activity was measured by hydrolysis of FDA, while the activity of CAT was determined by a respirometric technique, both methods reported by Amariei et al., 2017 in [30].

### 2.4. Cell Damage and Image Processing

**Scheme 2:**
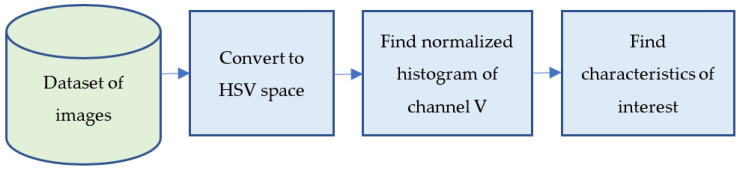
General block diagram of image processing procedure.

The quantitative analysis of confocal images to determine the number of live and dead cells, is usually performed manually. In this work, we applied, for first time, image processing techniques to search for characteristic parameters of the microscopic images that are proportional to the number of living and dead cells. A block diagram of the procedure used is presented in Scheme 2. Each stage of this procedure is described in dep in following Section 2.4.1, Section 2.4.2, Section 2.4.3, Section 2.4.4, Section 2.4.5.

#### 2.4.1. Dataset of Images

During the exposure test, samples of each reactor were taken and centrifuged for the separation of the solid fraction (arrow 3 in Scheme 1). The supernatants collected were used for cell staining and images capture by confocal microscopy.

Cell damage was evaluated using Live/Dead Backlight Bacterial Viability staining and laser confocal microscopy (CLSM), Leica TCS-SP5, Wetzlar, Germany. The SYTO9/propidium iodide (PI) in DMSO was incubated with the samples following the manufacturer’s instructions. Viable cells were marked in green by SYTO9 (λex from 488 to 499 nm; λem = 540 nm), while non-viable cells were marked in red by PI (λex from 561 to 577; λem = 645 nm). 

For the automatic analysis, a dataset including one to four images of each stained sample was created. The image selection for the dataset was conducted based on those that were brightest and had the best focus.

#### 2.4.2. Convert to HSV Space

Despite being in colour (RGB), confocal images only contain information in one of the channels. The green channel shows viable cells, which are marked by SYTO9, while the red channel shows dead cells marked by PI. All images are square, with a size of 1024 × 1024 pixels, representing an area of 390 μm^2^ but they do not show bacteria in detail, only coloured points. Due to their variability, the analysis of clusters of bacteria (shape and size) was not considered adequate, opting to analyse the quantity and luminance or brightness of the bacteria in the images. The perception of brightness by the human eye means that a red object appears less luminous than a green one, even at the same saturation and lighting conditions. To solve this problem, all the images were changed from the RGB colour space (red, green, and blue colours) to HSV colour space (hue, saturation, luminance). This permitted us to compare the brightness of the images directly, without considering how the colour is perceived by the human eye, which could be a subjective source of error when manually counting the number of green and red cells in the image.

#### 2.4.3. Find Normalized Histogram of Channel V

Once the original images were transformed into HSV space, the histograms of the brightness channel (V) were obtained. Channel V has a dimension of 8 bits and each pixel can take values from 0 to 255. In the histogram, the occurrence frequency of each brightness value was obtained.

On the other hand, images have large areas without information, because bacteria tend to form clusters and cells was not uniformly distributed in field. To compare images with different bacterial densities, they were normalized to the zero-brightness value, that is, all elements of the histogram are normalized to the number of dark pixels (zero-brightness). See examples in Figure S2.

#### 2.4.4. Find Characteristics of Interest

With the normalized histograms, the number of pixels corresponding to viable bacteria (images obtained with the green marker) and the number of non-viable bacteria (images obtained with the red marker) were determined. Empirically, the maximum value of 127 bins histogram was used to obtain the viable/non-viable cell ratio. However, in tests conducted using other parameters such as the sum of all the values of the histogram or by using different numbers of bins, the results were similar.

#### 2.4.5. Software Used for Image Processing and Statistical Analysis of Results

Confocal images were analysed using Matlab R2021a (The Mathworks, Inc., Natick, MA, USA). A principal Component Analysis (PCA) was conducted using Statgraphics 19 (Statgraphics Technologies Inc., The Plains, VA, USA). 

## 3. Results and Discussion

The experimental measurements of chemical parameters, toxicological and biochemical information collected from enzymatic activities, as well as cell viability, are presented and analyzed below.

### 3.1. Effect of PPCPs on Organic Matter Removal and Biogas Production

**Figure 1:**
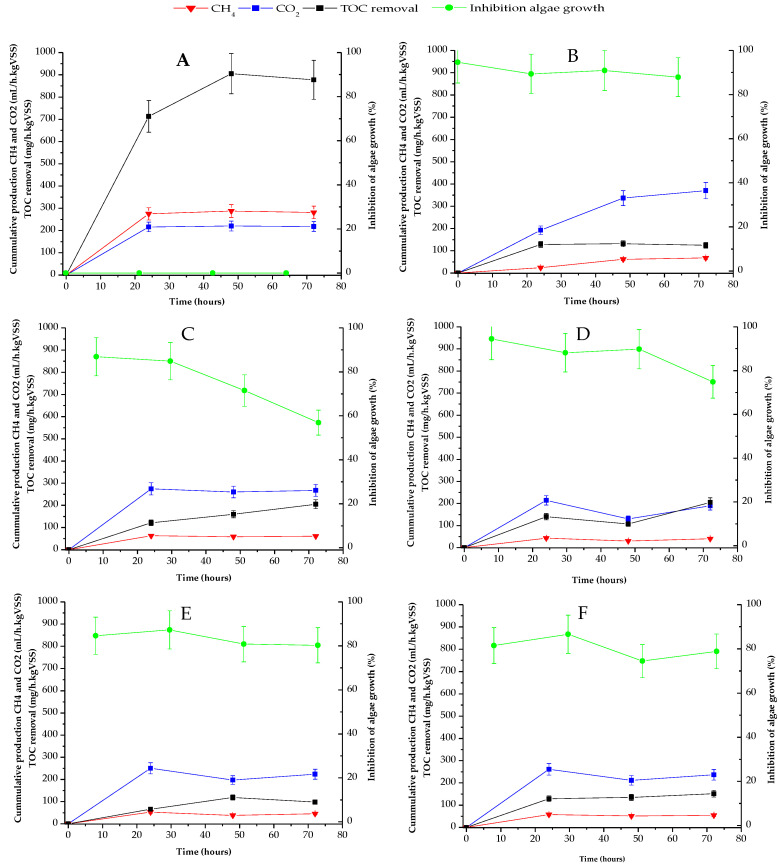
Organic matter removal (in black squared lines), biogas production (in red triangular and blue rhomboidal lines) and ecotoxicity (in green circled lines) in anaerobic reactors: (**A**) Control reactor without pollutants; (**B**) Reactor containing SFM; (**C**) Reactor containing CBM; (**D**) Reactor containing IBU; (**E**) Reactor containing TCS; (**F**) Reactor containing mixed pollutants.

Figure 1 shows the variation profile of the organic matter removal rate (in black squared lines), production of methane (in red triangular lines), and carbon dioxide (in blue rhomboidal lines), as well ecotoxicity (in green circled lines), along exposure time, in all anaerobic reactors used. 

Here, it can be observed that the best performance appears in the reactor without PPCPs (Figure 1A). This reactor reached 875 mg/h kgVSS of TOC biodegradation at 72 h of exposition time, coupled with a constant biogas production with a methane content slightly higher than CO_2_. Regarding the toxicity, the liquid of the control reactor did not inhibit algae growth along the time. 

In contrast, the TOC removal rate was negatively affected by the presence of individuals PPCPs and their mixture, as can be seen in Figure 1B–F. TOC removal rates barely reached 200 mg/h kgVSS at 72 h in anaerobic reactors containing CBM and IBU, while in systems with SFM, TCS and their mixture, organic removal rates were below 100 mg/h kgVSS. These values show a decay in the removal rates when compared with the ones from the control reactor, at the same exposure time, of 77%, in presence of CBM and IBU (Figure 1C,D) and 88% in the reactor with SMF, TCS and mixed pollutants (Figure 1B,E,F).

Methane production was also negatively affected by PPCPs, showing a similar behaviour to that of TOC removal. With regard to Figure 1A, the control reactor produced 300 mL/h kgVSS of methane at 72 h, but in the presence of PPCPs, the methane generation rate was lowered to 50 mL/h kgVSS at the same time (Figure 1B–F). These values correspond to 83% inhibition of methanogenic activity in the bioreactors containing pharmaceuticals when compared to the one in the control system.

Interestingly, carbon dioxide production was favoured over methane generation in all reactors, suggesting a short circuit between acidogenic and methanogenic steps in the anaerobic digestion process. The CO_2_ production rate was maintained at near to 200 mL/h kgVSS in almost all reactors, including the control one. However, in presence of SFM the generation of carbon dioxide was stimulated, reaching 400 mL/h kgVSS, which is twice the production rates obtained in the other bioreactors.

Some previous works also evaluated the effect of several pharmaceuticals on AD, where attention is specially focused on biogas production, but not to organic matter removal. In this manner, it was proven in the literature that IBU inhibits acetoclastic methanogens but does not cause inhibition to other trophic groups involved in the anaerobic process [49]. In fact, in that work it was stated that hydrogenotrophic methanogens were not affected by IBU. Besides, in [50,51] nonnegative effects of IBU on AD were evidenced, at concentrations of up to 206 mg/L. However, the works referred to were conducted using acclimated biomass, which showed an adaptive response to the anti-inflammatory drug.

The effect of TCS on AD was also analysed in [52,53]. In their study, the authors agree that TCS decreases methane production, which was more apparent with an increasing concentration of the antibacterial compound, and even altered the microbial ecology of the anaerobic reactors. 

The impact of nine individual and mixed pharmaceuticals on long-term biogas production, comparing their effect in adapted and non-acclimated sludge was also evaluated in [54]. The authors state that antibiotics and psychostimulants (i.e., CBM) were the main causes of the inhibition processes observed in adapted biomass but could be stimulatory for biogas production when using non-adapted biomass. In addition, the mixture did not present a clear different effect compared to that produced by individual pollutants, so the authors explained the experimental result based on the adaptive response of the microbial population.

From the analysis of the published literature, it can be proposed that the response of anaerobic biomass to pharmaceuticals is dissimilar and very difficult to compare, due to the different operational conditions, contact time, inoculum used, and the type and concentrations of micropollutants in the experiments, i.e., the same compound may be reported as harmless for the anaerobic bacteria in one work, whereas it can lead to imbalances in AD in another one.

Regarding the ecotoxicity evaluation, the results included here show that the effluents of anaerobic reactors containing individuals PPCPs, and their mixture were very toxic to algae populations initially, showing inhibition >90% for SFM and IBU, and >80% for CBM, TCS and the mixture of pharmaceuticals. Unfortunately, these results are difficult to compare with previous works, due to the lack of information on the effluents’ toxicity of anaerobic reactors in treating PPCPs there. The only article found that focused on this point was the work of Ji et al. [20]. In their work the individual and mixed acute toxicity of AD intermediates (acids and alcohols) and some antibiotics (polymyxin, aureomycin and chloromycetin) were analysed using the luminescent bacterium *Vibrio fischeri*. The authors of [20] found that the intermediates compounds and antibiotics caused toxicity that affect AD. The joint toxicity was also evaluated in their work, resulting in different behavior between intermediates and drugs. The authors finally determined additive or synergistic effects, depending on the combined compounds studied. In the present work, detoxification was only observed in reactors with CBM and IBU (Figure 1C,D). For these two systems, the ecotoxicity was lowered to 58% inhibition (with CBM) and 74% (for the IBU) at 72 h of cultivation in both cases. Obviously, the ecotoxicity measured in the bioreactors is linked to the presence of the PPCPs parents, but it must also be analysed in relation to the metabolites generated by the co-metabolism of the organic matter. 

**Figure 2:**
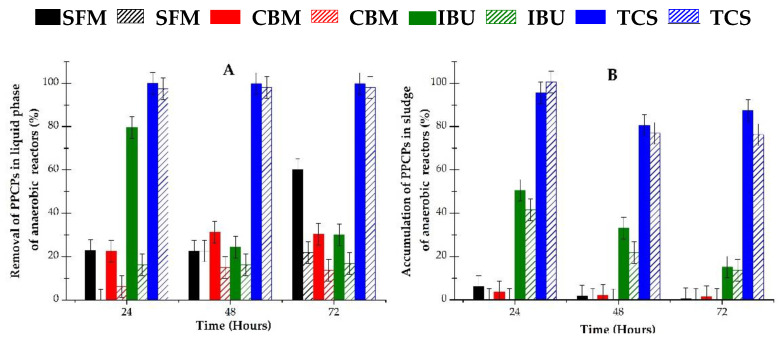
Removal of selected pollutants in aqueous media and their accumulation into the sludge of anaerobic reactors: (**A**) liquid fraction; (**B**) solid fraction (sludge). Filled bars correspond to individual contaminant in reactors; hatched bars correspond to mixture of pollutants in reactor.

Metabolites of PPCPs are usually unknown, but their presence can be estimated from the mass balance of each measured compound. Figure 2 shows the balance of PPCPs in the liquid and solid fractions of the anaerobic systems used. The analytical parameters of the HPLC-DAD method that were used, and a typical chromatogram obtained from the experiment are shown in Table S1 and Figure S1 (Supplementary Materials). The analytical method presents a high linear correlation for all contaminants, and also a good resolution for the four peaks detected.

As can be seen in Figure 2, different profiles of PPCPs were obtained in the liquid phase of the anaerobic reactors. Nevertheless, a clear connection is observed between both phenomena, namely, the removal of pharmaceuticals in the liquid fraction and their accumulation in the sludge.

IBU (filled green bars in Figure 2A) appears as highly biodegradable at 24 h, showing 80% removal in the liquid phase, though the removed pollutant was partially accumulated in the sludge. In fact, as can be seen in Figure 2B, almost 50% of the initially added mass of IBU was found in the solid fraction of the bioreactor. According to these data, only 30% of the IBU was thus effectively metabolized by the anaerobic biomass. In addition, the IBU content in liquid decreased with time, but it did not appear in the solid fractions, meaning that it remained unaltered in the solution and was partially absorbed in the sludge.

SFM and CBM demonstrated similar behavior in liquid and solid fractions, as both contaminants were found to be medium or poorly biodegradable, respectively. Their removal varies with time, from 20% to 60% for SFM (filled black bars in Figure 2) and from 20% to 30% for CBM (filled red bars in Figure 2). A low concentration of these compounds was detected in sludge, indicating that it remained untransformed in aqueous solution, or bio transformed, especially at longer incubation time.

A completely different behavior was found for TCS (filled blue bars in Figure 2). This compound was almost completely removed from the liquid, but it was retained in the sludge and untransformed.

Regarding to the behavior of contaminants in mixtures, which correspond to the hatched bars in Figure 2, a similar trend to that shown by individual contaminants was observed. In general, lower removal of mixture in liquid phase was detected for all compounds. However, only IBU and TCS were detected in these reactors’ sludge, as neither SFM nor CBM were found in significant quantities in their solid fraction.

Recently [43], the biotransformation degree of several organic micropollutants in the AD process was compared. Based on the published data, the authors classify these contaminants depending on their removal yields. According to the information collected in [43], SFM presents a high removal yield (≥75) under anaerobic conditions; IBU and CBM have low removal yields (≥25%) in the same conditions; and TCS medium-low behavior (from 25% to 50%). These values agree with the results presented in this work.

The biological removal of selected PPCPs is a very complex process, especially in anaerobic reactors, in which different bacterial trophic groups are involved. As an example, the anaerobic removal of SFM was carried out by methanogens, acetogens, sulfate reducing bacteria and others [55,56]. This phenomenon, added to the different physicochemical properties of PPCPs, makes it difficult to explain the performance of anaerobic reactors under the pressure of micropollutants. However, most microorganisms have similar enzymatic systems, so to better understand the biotransformation, it is probably easier to validate the enzymatic activity of the whole biomass.

### 3.2. Enzyme Response to PPCs

**Figure 3:**
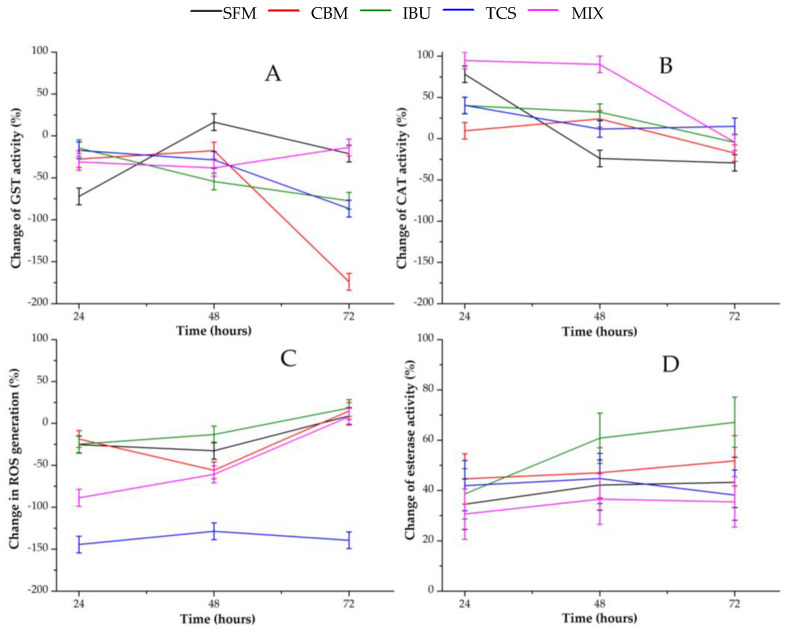
Profile of enzymatic activities of anaerobic biomass exposed to selected PPCPs: (**A**) GST activity; (**B**) CAT activity; (**C**) ROS generation; (**D**) Esterase activity.

Figure 3 shows changes in enzymatic activities along time for all PPCPs containing bioreactors. Activity variations were calculated with respect to those in the control reactor at the same time. Therefore, negative variations denote a hyper-expression of the corresponding enzyme in presence of the pollutants. In contrast, positive variation values indicate the inhibition of the corresponding enzyme.

With regard to Figure 3A, which represents changes in GST activity, a clear hyper-expression of this detoxification enzyme is evident, especially in reactors with CBM, TCS and IBU, at 72 h. In the reactor with mixed pollutants, minor alterations were detected, while in the one with SFM an inhibition peak appears for GST activity at 48 h.

Regarding the antioxidant enzyme CAT, its initial behavior was completely different to that of GST. According to Figure 3B, CAT was completely inhibited after 48 h of exposure to the mixtures of pollutants. Individual PPCPs initially caused lower degree of inhibition in CAT activity. However, this enzyme returned to its normal level, in the CNT reactor at the end of the experiment.

Figure 3C shows the profile of ROS generation, resulting in a clear accumulation of oxidative species in all PPCP-containing reactors compared to that in the CNT-containing one. ROS generation caused by TCS initially was higher than its values in the other digesters. On the other hand, oxidative stress decreased in all bioreactors containing PPCPs, approaching the normal level at 72 h, except for TCS system in which it was maintained.

A change in esterase activity is shown in Figure 3D. Here, a moderate to high inhibition of this hydrolase can be observed for all systems. A higher inhibition was obtained in the IBU reactor, increasing with the exposure time.

On the other hand, the activities of both enzymes associated with oxidative cell damage were similar. In fact, the activity of GST and CAT increased with time. These profiles can be associated with ROS generation.

In a previous work, we reported ROS overproduction in aerobic sludge due to TCS and IBU [26]. This was accomplished with the higher activity of GST along time, also in aerated reactors. Changes in esterase activity in the presence of IBU, TCS and their mixture in aerobic bioreactors were also stated in our previous works [26,30]. Here, it was observed that esterase activity was inhibited to a lesser extent (<15%) or not affected by the presence of the organic pollutants. These previous results cannot compared with those obtained in this work, because the microbial community and its metabolism were different, as well as the reactor configurations and operational conditions. However, the response to oxidative stress of living organisms is quite similar at the cellular level. 

The role of enzymes in the anaerobic degradation of several organic micropollutants was also previously studied [57,58]. In previous studies, the key enzymes of oxidoreductases, transferases, hydrolases and lyases were analyzed, and were found to remove some PPCPs. Most recently [59], it was reported that the antidepressant fluoxetine inhibited key enzymes involved in hydrolysis of proteins and polysaccharides in anaerobic sludge stabilization process. Unfortunately, the PPCPs studied in these previous works were different to those evaluated in our work. Nevertheless, it has been demonstrated that different enzymes are activated or inhibited depending to the contaminants in anaerobic reactors and on the changes in operational conditions. However, to the authors knowledge, no work has studied the connections between alterations of the enzymatic activities and the anaerobic biomass cell viability.

### 3.3. Evaluation of Cell Damage and Cell Viability 

In the live/dead test, the viable bacteria and those that were non-viable were marked with the reagents SYTO9 and PI. Table 1 shows images obtained for the control reactor and the TCS containing reactor at 24 h of exposition. In the first line of this table, green and red cells marked by the double staining can be observed. The second and third lines in Table 1 show images transformed to HSV colour space, which is the basis of analytical image processing. 

According to the images in Table 1, the total number of pixels with information (whether green or red) is evaluated and compared to the black background regardless of their luminosity. That is, the reagent marks the state of the bacterium with two possibilities, i.e., alive or dead, without intermediate gradation, so for the extraction of results, a very bright pixel is considered as often as a dimly bright one.

Automatic image processing for the quantitative analysis of living cells in anaerobic reactors is not usual. In fact, quantitative analysis of images from CLSM have usually been conducted in medicine and biochemistry. Images are often used as qualitative and quantitative evidence of the phenomena observed at the cellular level, also to detect morphological changes at a single cell level, bacterial clusters and biofilm formation using software supplied by the apparatus manufacturer or free software [60,61]. However, it some references to biomass image analyses can be found in the literature. Melo et al., in 2021 [62], used quantitative image analysis techniques for the characterization of morphological alterations in microbial aggregates due to the presence of IBU in aerobic treatment reactors. However, the procedure and technique used in this previous article were completely different to those carried out in our work. The aim of Melo et al., 2021 was to evaluate alterations of the size and morphological structure of microbial flocs, which are relevant characteristics for the sedimentation process in aerobic wastewater treatment, not to determine the number of living cells in flocs.

**Figure 4:**
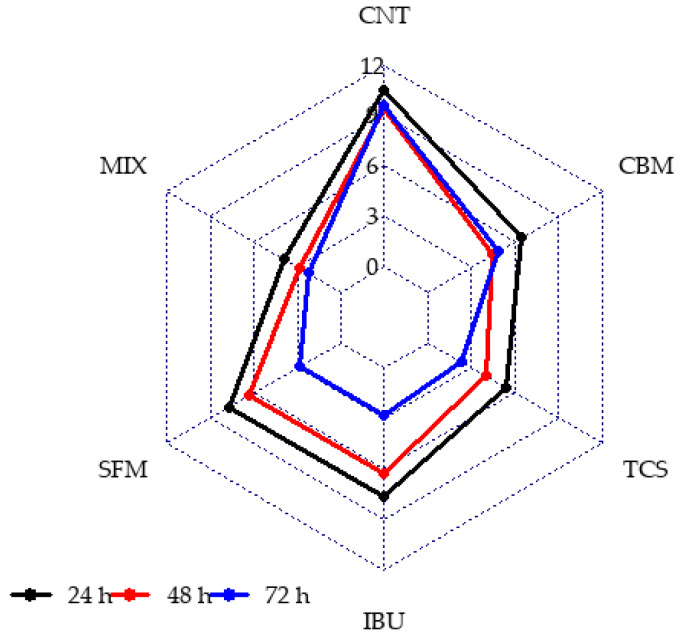
Viable to non-viable cell proportion in anaerobic reactors in presence of PPCPs at different culture time.

The information obtained from image processing was correlated to the exposure time and PPCPs in bioreactors. Figure 4 shows the radial chart of viable to non-viable cells proportion at 24, 48 and 72 h of reactions for each bioreactor studied.

In Figure 4, it can be observed that the CNT reactor presents higher values of viable/non-viable cells ratio, which denotes minor stress level and a healthy microbial population, regardless of culture time. In contrast, for PPCPs containing reactors, variation profiles for all contaminants were observed throughout the duration of operation. Lower values of viable to non-viable cells were obtained for TCS and MIX reactors. In contrast, SFM and CBM showed a similar ratio and higher values than that obtained in the TCS and MIX reactors. In all cases, the ratio decreased with time.

### 3.4. Correlation of Chemical and Biochemical Information Obtained in Anaerobic Reactors

**Figure 5:**
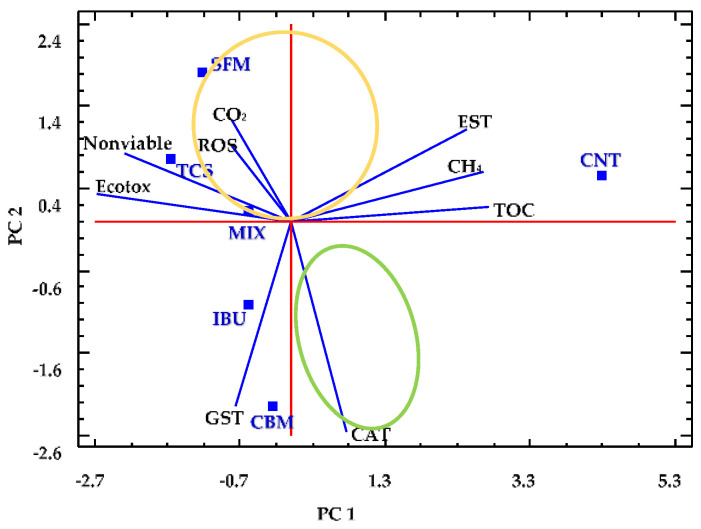
Principal component analysis (PCA) for anaerobic digesters. Squares represent the bioreactors containing PPCPs (SFM, TCS, IBU, CBM, MIX) and the reactor free of pollutants (CNT). Lines indicate the parameters measured, including chemical (TOC, CH_4_, CO_2_) and biochemical (EST, ROS, GST, CAT, nonviable cell fraction and Ecotox).

Statistical analyses of complex systems, in which a great number of parameters are measured, are necessary to identify relevant variables, which help to better understand the observed phenomena. For this, we used PCA to integrate all of the information collected from the anaerobic reactors. Figure 5 corresponds to the biplot resulting from PCA of data after 72 h of reaction.

In that last analysis, we selected the following nine numerical variables: TOC removal, (TOC); cumulative methane production (CH_4_); cumulative carbon dioxide production (CO_2_), ecotoxicity (Ecotox); all enzymatic activities measured (EST); ROS generation; and the fraction of nonviable cells obtained (Nonviable). The values of all selected variables were normalized by subtracting their mean and dividing them by their standard deviations.

Two principal components were then determined, namely, PC1 and PC2, which explain 75% of the variance, with eigenvalues of 4.69 and 2.05, respectively. The variables TOC, CH_4_ and EST correlated positively with PC1, showing a strong relationship between them. However, the variables Ecotox and Nonviable are negatively correlated with PC1 but strongly related between them. In contrast, CO_2_ and ROS have great relevance in PC2 and strongly related between them. PC2 are also negatively affected by CAT and GST, which are also strongly related.

In Figure 5, it can be observed that the performance of the control reactor can be well described by PC1. In fact, the CNT reactor presents higher values of TOC removal, CH_4_ production and esterase activity when compared to the values from PPCPs containing reactors, which appear at the opposite region of the CNT in the biplot. In addition, CNT reactor presented low values of ROS generation and ecotoxicity.

On the other hand, TCS and MIX reactors have similar performance, both characterized by high ecotoxicity and nonviable cells values. Next to TCS and MIX reactors, the SFM reactor appears, which presents a strong correlation between ROS generation and CO_2_ production. These three anaerobic systems containing PPCPs can thus be grouped due to their similar behaviors (see yellow ellipse in Figure 5).

Similarly, the performance of CBM and IBU reactors (grouped in a green ellipse) can be explained by the variables GST and CAT. For these systems, the low values of GST and CAT have a great influence on the reactors performance.

## 4. Conclusions

An evaluation of the impact of the single pharmaceuticals and their mixture was achieved for the anaerobic digestion process. According to the results obtained in anaerobic batch reactors, the SFM, TCS and mixed organic pollutants had higher negative impact on the organic matter removal, compared to CBM and IBU. Similar behavior was observed for cumulative methane production, while SFM stimulated carbon dioxide production.

High ecotoxicity values were obtained at the initial time (between 90% to 80%) for all reactors, resulting in a slight detoxification for CBM and IBU. The toxicity observed was associated with the incomplete removal of pharmaceuticals compounds, which remains untreated in liquid phase of digester and retained by sludge.

In our work, the monitoring of enzymatic activities demonstrated the hyper-expression of the biotransformation enzyme GST, especially in reactors with CBM, TCS and IBU, while the antioxidant enzyme CAT was initially inhibited. ROS was clearly accumulated in presence of the pharmaceuticals, especially TCS. In contrast, esterase activity was mainly affected by IBU.

The evaluation of cell viability by automatic image processing techniques allowed us to determine that the viable/non-viable cell ratio decreased in the presence of all individual pharmaceuticals and their mixtures. The worst-case scenario has been detected for TCS and the mixture of pollutants, increasing dead cells along culture time.

Finally, combining the information the enzymatic activities, cell stress, chemical analysis, and cell viability a clear relationship between the pollutants SFM, TCS and MIX with higher ROS accumulation and higher toxicity level in reactors was found, both promoting the increment of CO_2_ production and cell death.

More studies are needed for a more in depth understanding of the complex relationships between trophic groups in anaerobic reactors. Special attention should be paid to the enzymes involved in the regulation of oxidative stress and detoxification process, which are probably better indicators of the process unbalances than the alterations of the microbial populations usually evaluated in bioreactors.

## Figures and Tables

**Table 1:** Confocal image and their processing for evaluation of viable and non-viable cells.

Image Treatment	CNT Reactor 24 h	TCS Reactor 24 h
Double staining (SYTO9/PI)	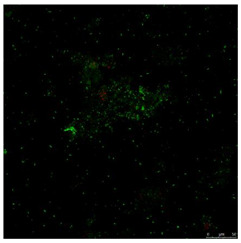	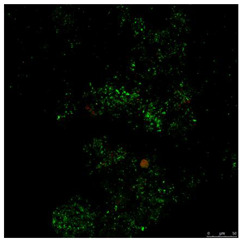
Luminance in green channel	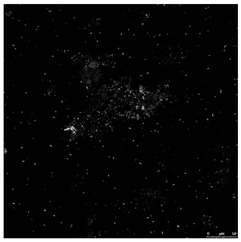	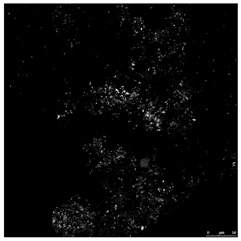
Luminance in red channel	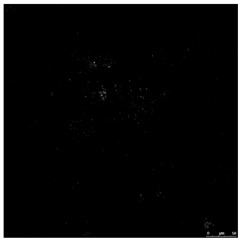	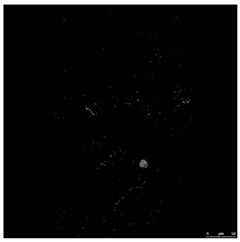

## Data Availability

Not applicable.

## Supplementary Materials

The following supporting information can be downloaded at: https://www.mdpi.com/article/10.3390/toxics10010042/s1. Table S1: Analytical characteristics of the method for the determination of selected PPCPs in anaerobic reactors, Figure S1: Chromatogram of standard mixture of PPCPs and sample of liquid in MIX reactor, Figure S2: Histogram of channel V for a control image. (a) 256 values, (b) 127 bins.
