# Supplementary material for: Effect of Carbamazepine, Ibuprofen, Triclosan and Sulfamethoxazole on Anaerobic Bioreactor Performance: Combining Cell Damage, Ecotoxicity and Chemical Information
Source: Toxics. 2022 Jan 17;10(1):42. doi: 10.3390/toxics10010042 (PMC8779021; doi:10.3390/toxics10010042)
Supplement: Supplementary file 1 [file toxics-10-00042-s001.zip › toxics-1507108-supplementary.pdf]

toxics

Effect of Carbamazepine, Ibuprofen, Triclosan and Sulfamethoxazole on Anaerobic Bioreactor Performance: Combining Cell Damage, Ecotoxicity and Chemical Information

Mabel Díaz-Cubilla, Pedro Letón, Carlos Luna, Marta Marrón-Romera and Karina Boltes

Table S1. Analytical characteristics of the method for the determination of selected PPCPs in anaerobic reactors.

	SFM	CBM	IBU	TCS
Elution time (min)	5.6	6.9	10.8	12.5
Linear range (mg/L)	1–10	1–10	1–10	1–10
r	0.97	0.98	0.97	0.97
LOD (mg/L)	2.75	2.69	2.72	2.73
LOQ (mg/L)	9.17	8.96	9.06	9.09

LOD (limit of detection); LOQ (limit of quantification).

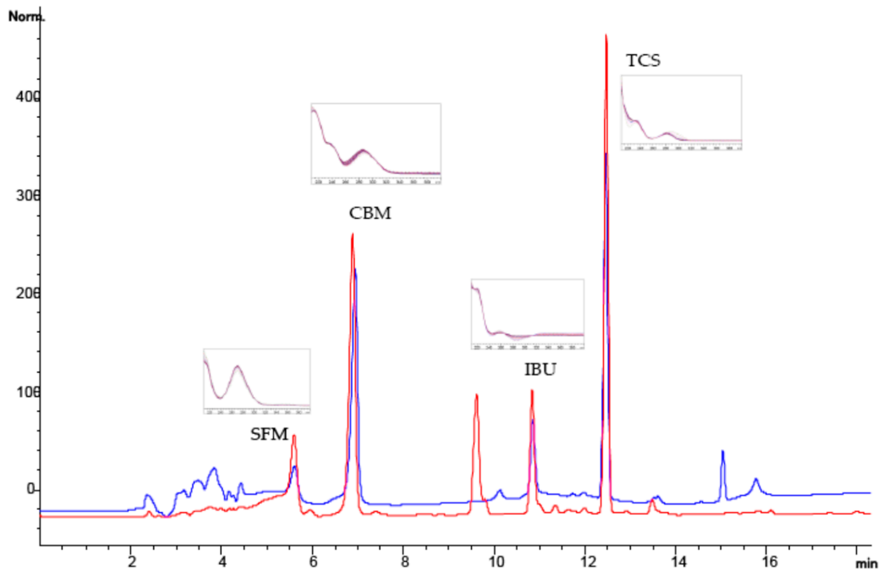

Figure S1. Chromatograms of standard mixture of PPCPs (blue line) and concentrated liquid sample of MIX reactor (red line). The names of pharmaceuticals and their spectrum of UV absorption are shown over the maximum of each peak.

As an example, Figure S2a) shows the normalized histogram channel V for a control image, and it can be seen that the highest concentration of information is in the low-brightness area of the histogram. To facilitate the interpretation of the histograms, the first two brightness values were eliminated and the data were grouped into 127 batches (or bins) of two values each, see Figure S2b).

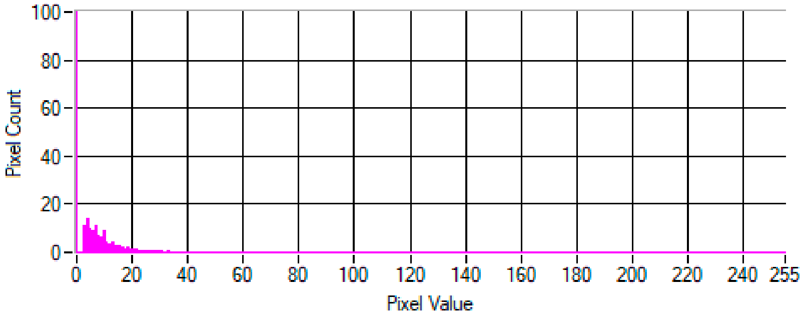

a)

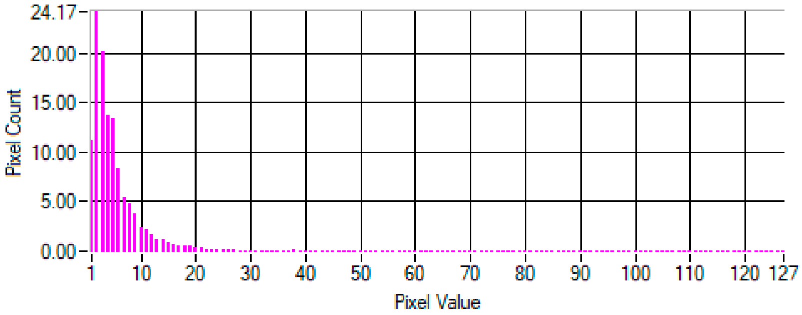

b)

Figure S2. Histogram of channel V for a control image. a) 256 values, b) 127 bins.
